# Diagnostic odyssey of patients with the rare immunodeficiency activated PI3 kinase delta syndrome (APDS): case study from expert and patient surveys

**DOI:** 10.3389/fimmu.2026.1763482

**Published:** 2026-03-04

**Authors:** Hermann Maximilian Wolf, Fabio Candotti, Roman Spelsberg, Georgios Sogkas, Hans-Holger Bleß, Kirsten H. Herrmann, Fabian Hauck

**Affiliations:** 1 Faculty of Medicine, Sigmund Freud Private University Vienna (SFU), Vienna, Austria; 2 Clinical Immunology Office, Vienna, Austria; 3 Division of Immunology and Allergy, Lausanne University Hospital and University of Lausanne, Lausanne, Switzerland; 4 fbeta GmbH, Berlin, Germany; 5 Department of Rheumatology and Immunology, Hannover Medical School (MHH), Hannover, Germany; 6 Pharming Group NV, Leiden, Netherlands; 7 Division of Pediatric Immunology and Rheumatology, Department of Pediatrics, Dr. von Hauner Children’s University Hospital, Munich Ludwig-Maximilians-Universität, München, Germany

**Keywords:** activated PI3K delta syndrome (APDS), diagnostic delay, inborn errors of immunity, patient journey, primary immunodeficiency, targeted therapy

## Abstract

**Introduction:**

Activated phosphoinositide 3-kinase delta syndrome (APDS) is an inborn error of immunity first described in 2013. With an estimated prevalence of 1–2 per 1,000,000 individuals, it is considered an ultra-rare disease. The aim of this survey was to explore the diagnostic and therapeutic challenges of patients with APDS from the patients` and physicians` perspective in Austria, Germany, and Switzerland.

**Methods:**

A qualitative case study approach was applied. Semi-structured interviews were conducted with six patients or legal guardians of children with APDS, and four clinical immunologists with direct experience in APDS care. Transcripts were analyzed using inductive content analysis.

**Results/discussion:**

The interviews revealed a median diagnostic delay of several years, mainly due to the rarity and phenotypic heterogeneity of APDS and the involvement of multiple specialties prior to referral to an immunologist. Many patients initially received symptomatic treatment before an underlying immune disorder was suspected. Physicians emphasized the decisive role of genetic testing for confirmation, while patients frequently described the diagnosis as a “lucky coincidence”. Both groups highlighted structural barriers including limited awareness, fragmented care, and delayed access to targeted therapy. Early recognition of APDS requires specific education across specialties, wider access to genetic testing, and the development of standardized diagnostic and disease activity tools. Strengthening interdisciplinary care pathways and timely initiation of APDS-specific therapy may substantially improve outcomes in this ultra-rare immunodeficiency.

## Introduction

Activated Phosphoinositide 3-Kinase Delta Syndrome (APDS) is a rare inborn error of immunity (IEI) first described in 2013 (1, 2). Also referred to as PASLI (PI3K-p110δ activating mutation causing senescent T cells, lymphadenopathy, and immunodeficiency), it arises from activating mutations in the PI3Kδ pathway that cause senescent T cells, lymphoproliferation, and immunodeficiency. APDS is now recognized as part of the expanding spectrum of inborn errors of immunity, with more than 500 entities described to date (3). Despite increasing awareness, the heterogeneous clinical presentation and rarity of APDS often result in diagnostic delay, with patients frequently diagnosed years after initial symptom onset (4). Timely identification, however, is critical, as early treatment may prevent or attenuate irreversible organ damage.

Clinically, APDS manifests through impaired immune cell function and recurrent infections. Age of onset varies from early childhood to adulthood (5). Disease expressions are broad, encompassing recurrent sinopulmonary infections, lymphoproliferation, autoimmunity, enteropathy, and lymphopoietic malignancies. In a systematic review of 243 cases, pneumonia was reported in 43.6% of patients, otitis media in 28.8%, and sinusitis in 25.9%. Immune dysregulation was also common, including lymphoproliferation (70.4%), autoimmunity (28%), enteropathy (26.7%), growth failure (20.6%), and malignancy (12.8%) (6).

Diagnosis relies on clinical suspicion informed by history, examination, and family background, with confirmation by genetic testing. However, no single biomarker or pathognomonic feature exists. Consequently, APDS patients often undergo extensive evaluation across multiple specialties before receiving a molecular diagnosis. Median age at diagnosis has been reported at 12 years, with a median delay of 7 years after symptom onset (6). This prolonged and fragmented process, termed “diagnostic odyssey,” represents a major challenge in clinical practice.

Current therapeutic strategies for APDS remain limited. Besides allogeneic hematopoietic cell transplantation, there is no curative treatment, and most approaches focus on symptom control. Supportive management includes immunoglobulin replacement therapy, antimicrobial prophylaxis, and immunosuppressive drugs such as glucocorticoids or mTOR inhibitors (e.g., sirolimus) (4). In recent years, targeted inhibition of PI3Kδ has emerged as a promising therapeutic strategy. Leniolisib, the first-in-class PI3Kδ inhibitor, was approved by the FDA in 2023, followed by approvals in Israel and the United Kingdom in 2024, and Australia in 2025. Regulatory review by the European Medicines Agency is ongoing (7–10). These approvals represent a milestone in the management of APDS, although long-term outcomes remain under investigation.

Despite these advances, diagnostic challenges persist due to limited disease awareness and symptom overlap with more common diseases (e.g. common variable immunodeficiency). Patients are often initially treated for recurrent infections without recognition of the underlying immune deficiency. The lack of systematic diagnostic markers further complicates case identification. Improving awareness across diverse specialties, including pediatrics, internal medicine, otolaryngology, pulmonology, gastroenterology, rheumatology and hematology, will be essential for earlier recognition and referral.

Optimizing the diagnostic pathway is therefore critical for improving outcomes. Delayed diagnosis not only prolongs morbidity but also increases the risk of irreversible complications such as bronchiectasis, chronic organ damage, or malignancy. A more systematic approach is needed, encompassing early recognition of characteristic symptom clusters, streamlined access to genetic testing, and timely referral to immunology specialists.

Existing literature has focused primarily on registry-based analyses (11), systematic reviews of clinical and genetic features (6), or physician surveys addressing diagnostic challenges and therapeutic decision-making (12). While these contributions have advanced understanding of APDS, they rarely integrate patient perspectives. Yet, patient experiences provide crucial insights into delays, misdiagnoses, and psychosocial consequences of the diagnostic odyssey. Case reports and narratives published by patient organizations, such as the German association for congenital immunodeficiencies (13), highlight the importance of including the patient voice, but these remain anecdotal and lack systematic evaluation. To date, no multi-perspective qualitative study exploring diagnostic and therapeutic pathways in APDS within Austria, Germany and Switzerland has been published.

This health services study seeks to address this gap by reconstructing the diagnostic odyssey through retrospective surveys involving patients, legal guardians, and treating physicians. By mapping the pathway from initial symptom onset to confirmed diagnosis, the study aims to identify key barriers, delays, and physician groups most frequently involved in early care. Parallel evaluation of therapeutic strategies sheds light on current standards of care, decision-making processes, and access to emerging targeted therapies. The integration of physician and patient perspectives allows for a more comprehensive understanding of challenges in APDS management, ultimately supporting earlier recognition and more personalized treatment.

## Methods

To investigate the diagnostic pathway of patients with APDS within the healthcare systems of Austria, Germany and Switzerland, a qualitative case study approach was employed. The object or phenomenon of investigation in this case study was the diagnostic process of APDS. The intermediate context to which the case study was applied comprised patients diagnosed with APDS. The broader or more specific context was the diagnostic and therapeutic care provided within the healthcare systems of Austria, Germany and Switzerland. To analyze the phenomena across the different contextual levels, patient and physician surveys were conducted.

### Recruitment

According to the study protocol, physicians from Austria, Germany and Switzerland were recruited. These physicians contacted their patients and asked for consent to participate in the study. The dsai invited patients from their network to give their consent to participate in this survey.

### Data collection and analysis

Primary data were collected via patient and physician interviews. Physicians were identified through publicly available contacts and approached by the study coordinator (FH); comprehensive coverage of all relevant physicians could not be guaranteed. Patients were recruited through participating physicians and the patient organization dsai. Participation was voluntary, and interviews were audio-recorded with explicit consent. Data collection occurred via telephone or online platforms (Microsoft Teams).

Semi-structured interview guides, developed for this study by RS and validated by FH, were tailored for patients (or legal guardians) and physicians to ensure comparability while capturing group-specific perspectives. Both guides addressed diagnostic pathways, therapeutic management, and challenges in APDS care. Patient interviews focused on the individual diagnostic odyssey, consultations prior to diagnosis, symptom onset, confirmation of APDS, disease burden, daily life changes, involvement in decision-making, and perceived care challenges. Physician interviews explored professional experiences, including age and disease stage at diagnosis, diagnostic procedures, specialties involved, factors contributing to delay, therapeutic strategies, care coordination, patient engagement, and systemic challenges.

Interviews were analyzed using qualitative content analysis (14), augmented by elements from Naeem et al. (15). All interviews were transcribed with verbatim. Coding combined inductive and deductive approaches: inductive codes emerged from the data, while deductive codes were based on established theoretical frameworks. Text segments were assigned to thematic categories, clustered into higher-order themes, and interpreted to integrate individual experiences into broader conceptual patterns. MAXQDA software supported data management and coding (16). Key quotations were selected for their relevance to study objectives and research questions. In addition, selected clinical and diagnostic information reported during the interviews were systematically extracted and summarized to provide a structured overview of individual diagnostic pathways.

### Ethical and regulatory considerations

The study was conducted in accordance with the Declaration of Helsinki and applicable legal regulations, including General Data Protection Regulation (GDPR). Ethical and legal approval was granted by the Ethics Committee of LMU Munich on November 27, 2024.

## Results

### Description of the interviewed population

The study included four physicians from Austria (1), Germany (2) and Switzerland (1), all experienced in pediatric and adult immunology, rheumatology, hematology, and/or oncology. Each held board certification in clinical immunology, in pediatrics or in internal medicine, complemented by subspecialty training in immunology, rheumatology, and/or pediatric hematology/oncology. All had extensive experience in managing IEIs, including APDS, with patient loads ranging from one to seven APDS-individuals per physician. Some clinicians also supervised patients retrospectively diagnosed following the initial characterization of APDS in 2013. Collectively, the physician sample represented a highly qualified cohort with longitudinal, cross-age expertise in APDS diagnosis and management across diverse clinical settings.

Six patients participated in the survey, comprising three adult patients and three legal guardians of children diagnosed with APDS. Recruitment occurred through the dsai network and direct physician contact. Participants were drawn from Austria (one adult patient) and Germany (2 adult and 3 pediatric patients). Despite outreach efforts in Switzerland, no patients from this region were enrolled. For three of the six patients included, legal guardians answered the interview questions.

### Summary of content analysis

Qualitative content analysis assigned codes to three overarching themes and their associated concepts, as summarized in **Table 1**.

**Table 1:** Topics and codes of the qualitative content analysis.

No.	Theme	Codes
1	Diagnosis	• Age at time of diagnosis • Symptoms at time of diagnosis • Time to diagnosis • Factors contributing to delayed diagnosis • Specialization of diagnosing physicians • Diagnostic procedures
2	Therapy	• Changes in disease experience • Therapeutic steps • Specialty of treating physicians prior to diagnosis • Specialty of treating physicians after diagnosis • Therapy decision-making • Monitoring of disease course • Time to initiation of therapy
3	Challenges	• Challenges in therapy • Challenges in diagnosis • Structural conditions required to address challenges in diagnosis and therapy

### Diagnostic pathways in APDS

Both patient and physician surveys revealed that the diagnostic pathway for APDS was characterized by clinical heterogeneity, evolving diagnostic technologies and varying levels of awareness among healthcare providers. **Table 2** provides a structured overview of the diagnostic pathways and treatment patterns reported by the six interviewed patients. Across cases, symptom onset occurred predominantly in childhood, while age at genetic confirmation varied widely, illustrating prolonged and heterogeneous diagnostic pathways. Participants reported that although molecular diagnostics had significantly advanced over the past decade, delays in diagnosis remained common. These delays were often multifactorial, involving nonspecific clinical presentation, limited disease recognition in primary care, and inconsistent access to specialized immunological evaluation.

**Table 2:** Overview of patient journeys and diagnostic timelines in six APDS cases.

Category	P1	P2	P3	P4	P5	P6
Country	Germany	Germany	Germany	Austria	Germany	Germany
Patient type	Pediatric	Pediatric	Adult	Adult	Pediatric	Adult
Age at symptom onset	Infancy (~8 months)	Early childhood	From birth	From birth	Childhood	Childhood
Key initial manifestation	Recurrent otitis media; streptococcal pharyngitis/scarlet fever; prolonged febrile episodes	Chronic cough; inspiratory breathing difficulties; failure to thrive; hoarseness	Neonatal respiratory distress; recurrent pneumonias and otitis media; growth difficulties; lymph node abscesses	Recurrent infections and chronic inflammatory symptoms	Therapy-refractory arthritis; immune dysregulation manifestations	Recurrent respiratory infections; pronounced lympha-denopathy
Major complications prior to diagnosis	Recurrent severe infections; suspected antibody deficiency	Lympho-proliferation with airway involvement	Chronic pulmonary involvement	Malignancy (Hodgkin’s disease)	Severe inflammatory disease course requiring hematopoietic stem cell transplan-tation	Cancer suspicion due to lympha-denopathy
First specialist(s) consulted	General pediatrics; Ear, nose and throat	Pediatrics; Ear, nose and throat; pulmonology	Pediatrics; pediatric hospital care	General practice; pediatric hospital care	Pediatrics; rheumatology/immunology care	Pediatrics; hospital-based care
Trigger for immunology referral	Abnormal immune parameters identified by new pediatrician	Bronchoscopy and abnormal immune findings	Antibody deficiency identified on blood work	Malignancy prompting diagnostic escalation	Hypogamma-globulinemia and refractory disease course	Suspicion of malignancy prompting tertiary-center evaluation
Age at genetic confirmation	3 years 10 months	4 years 4 months	5 years	~16–17 years	Late childhood	~11 years
Therapy prior to diagnosis	Repeated antibiotic treatment; symptomatic management	Steroids; inhalation therapy; long-term antibiotics	Symptomatic management; inhalation therapy	Symptomatic treatment; steroids	Anti-inflammatory and symptomatic treatment	Symptomatic management during oncologic evaluation

Reported time to diagnosis ranged from 3.8 to 73 years, with a median of 11 years. Patients and physicians consistently described the diagnostic process as a prolonged and emotionally exhausting journey. One legal guardian summarized: “We were in and out of hospitals for years. Nobody thought to check the immune system. Only when our new pediatrician ran more comprehensive tests did things move forward.”

Several interviewees referred to the eventual diagnosis as a “lucky coincidence”, often triggered by a physician change or a referral to a university center: “We were lucky to end up at a university center. Without that referral, we might still be waiting.”

**Figure 1:**
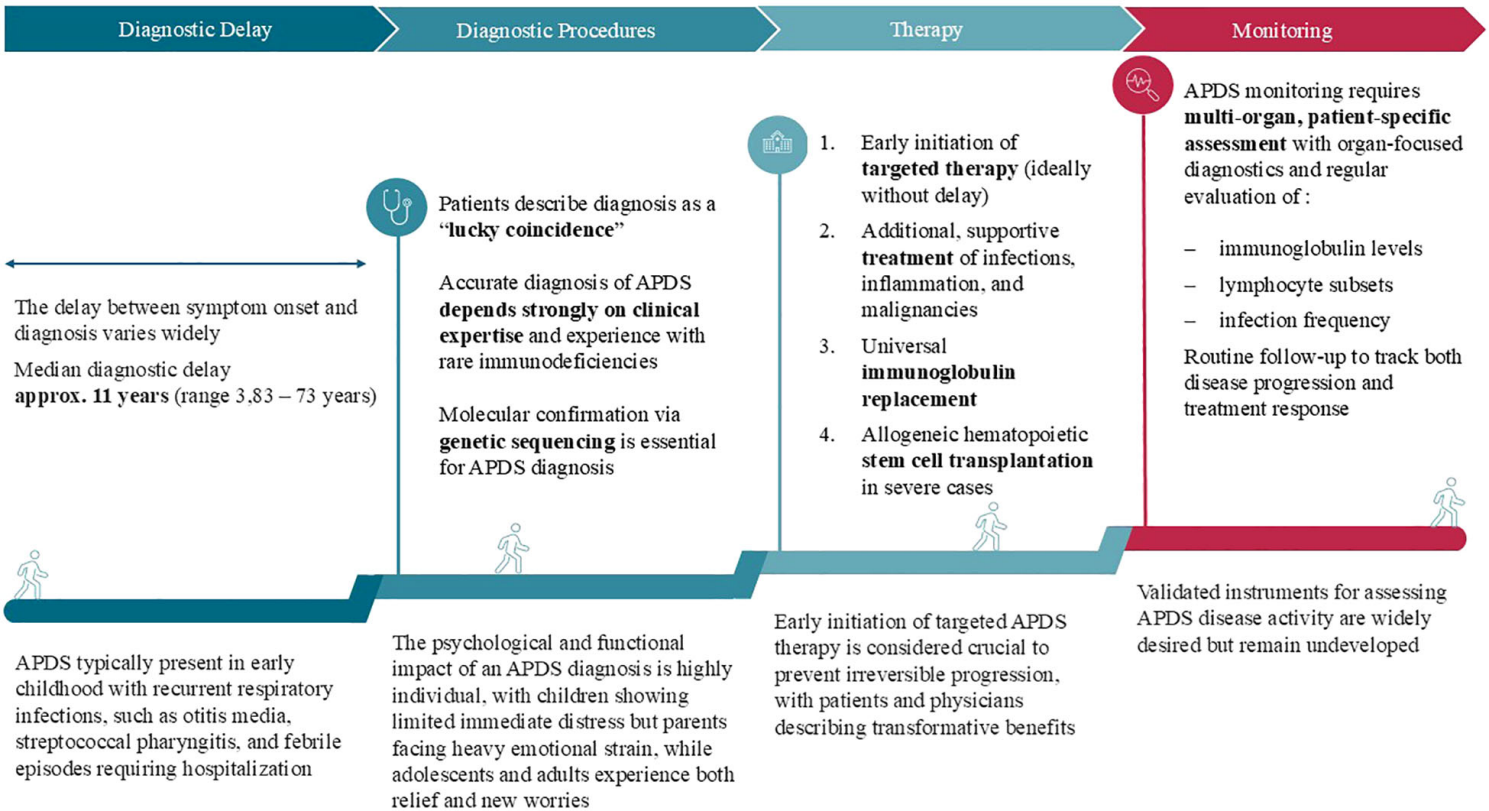
Summary of diagnostic and therapeutic pathway of patients with APDS.

The following **Figure 1** summarizes the diagnostic and treatment pathway of patients with APDS based on the results of the qualitative interviews.

### Diagnostic procedures

The interviewed physicians mentioned that APDS diagnosis required molecular confirmation via genetic sequencing of *PIK3CD* and *PIK3R1*. Clinical features, including lymphoproliferation, autoimmune cytopenia, or hypogammaglobulinemia, raised suspicion but were insufficient for diagnosis. Biochemical assays, such as PI3K phosphorylation analysis, remained largely confined to research settings. Interpretation of novel variants required interdisciplinary evaluation. Operational issues, including outsourced genetic sequencing and limited data accessibility, could delay confirmation. Patients highlighted the burden of the uncertainty remaining until the definite diagnosis is confirmed: “It was a fight. Every test result brought new questions. Only when we got the genetic result did things finally make sense.” Physicians advocated for centralized diagnostic pathways and improved integration of next-generation sequencing into routine practice.

### Barriers in diagnosis

During the interviews it was described that patients with APDS typically presented in early childhood with recurrent respiratory infections, including otitis media, streptococcal pharyngitis, and febrile episodes, which often required hospitalization. These infections could persist year-round and were accompanied by lymphoproliferation, autoimmune cytopenia, colitis, growth retardation, and pulmonary complications such as bronchiectasis.

Despite widespread technical availability of genetic testing, institutional, financial, and awareness-related barriers continued to impede timely APDS diagnosis. Patients frequently first presented to generalists or organ-specific specialists, including ENT (Ear, Nose, Throat) physicians, rheumatologists, or gastroenterologists, who often did not initially consider an IEI. Limited familiarity with rare diseases contributes to missed diagnoses: “With over 8,000 known rare diseases, even highly trained clinicians are likely to overlook conditions they have never encountered,” noted one physician. Both clinicians and patients emphasized the need for heightened suspicion when clinical courses deviated from expectations or standard therapies failed. Patients frequently reported prolonged dismissal or misattribution of symptoms, describing the diagnostic pathway as emotionally exhausting and information scarce.

Physicians highlighted variability in diagnostic standardization, citing limited access to functional assays, unclear interpretation of genetic variants of unknow significance, and institutional heterogeneity. Although genetic sequencing access and awareness had improved, cost and procedural inconsistencies remained substantial barriers.

### Impact of diagnosis

A confirmed diagnosis was considered as a turning point. It enabled targeted therapy and social reintegration. The psychological and functional impact of diagnosis varied by age and disease severity. Pediatric patients often adapted to restrictions early, while legal guardians experienced substantial emotional distress. Adolescents and adults perceived diagnosis as both a relief and a burden, which provided clarity but raised concerns about long-term complications. Physicians emphasized that definitive diagnosis stabilized patient self-perception and facilitated effective care. Patients and legal guardians echoed this and highlighted improvements in energy, infection control, and quality of life following initiation of immunoglobulin replacement therapy and other supportive measures. Nonetheless, structural complications such as bronchiectasis or inflammatory bowel disease could persist, necessitating ongoing multidisciplinary monitoring.

### Therapeutic management

Challenges for the management of APDS were attributed to the clinical heterogeneity of the disease but also to multiple systemic and structural challenges such as multiple specialists and lack of awareness for APDS and time to diagnosis. Management of APDS required an individualized approach, considering disease severity, organ involvement, comorbidities, patient preferences, and access to targeted therapies. Shared decision-making was described as central, with children above eight actively involved in therapeutic discussions. Treatment strategies were structured around five pillars: (1) initiation of APDS-specific targeted therapy, ideally early; (2) symptomatic management of infections, inflammation, or malignancy; (3) immunoglobulin replacement for antibody deficiencies; (4) allogeneic hematopoietic cell transplantation for severe or refractory cases; and (5) close surveillance for complications requiring additional immunomodulation. Timely initiation of targeted therapy was considered critical to prevent irreversible end-organ damage. Access remained limited by administrative barriers, although participants anticipated shorter delays as experience with therapy grew. During the interviews, patients emphasized the potential of targeted APDS therapy. One parent recalled: “After he started the therapy, he was like a different child. He ran, he was full of energy, we realized for the first time that life could be better.” Physicians confirmed the necessity to initiate targeted therapy as soon as possible: “We used to wait and rely on substitution therapy. But now, knowing the publications and based on my experience, I would initiate the targeted APDS therapy as early as possible.” In the form of a case study one physician reported: “For one patient, there was no perspective before the targeted APDS therapy. He was hospitalized with multiorgan disease, opportunistic infections, colitis and cytopenia. After starting therapy, he returned to work, gained weight, and no longer needed prophylactic medication.”

Targeted therapies had improved expectations for APDS care, yet clinical decision-making remained complex. Physicians faced challenges in determining the need for additional APDS-specific therapy in clinically stable patients and navigating administrative hurdles to access new medications. Patients and families reported daily treatment burdens and emotional stress, while simultaneously expressing relief at the availability of effective therapies.

### Monitoring and multidisciplinary care

Disease monitoring combined immunological parameters, organ-specific diagnostics, and patient-reported outcomes. Immunoglobulin levels, lymphocyte subsets, infection frequency, and functional biomarkers were assessed regularly. Pulmonary function, gastrointestinal status, and growth parameters were monitored according to individual organ involvement. Multidisciplinary teams, led by immunologists and including pediatricians, rheumatologists, gastroenterologists, pulmonologists, infectious disease specialists, and hematologists-oncologists, coordinated care based on dominant symptoms. Interdisciplinary case conferences and centralized communication were considered essential to optimize outcomes.

Interviews consistently identified a need for validated tools to assess APDS disease activity. Physicians suggested combining objective laboratory measures with patient-reported outcomes to capture the multisystem, variable phenotype of APDS. While disease activity scoring was partially implemented in the ESID registry, its application remained inconsistent. Such tools were viewed as valuable for clinical monitoring, prognostication, and therapeutic decision-making, though methodological challenges existed due to subjective symptoms and organ-specific complications. Patients similarly expressed the desire for clarity regarding disease progression and treatment evaluation.

### Challenges in diagnosis and treatment

APDS management was complicated by clinical heterogeneity and systemic barriers. Physicians strongly supported routine genetic testing and standardized disease activity assessment but expressed mixed views on the feasibility of current diagnostic procedures. Consensus emerged regarding the urgent need for approved, evidence-based targeted therapies, reflecting limitations in access outside compassionate use programs.

**Figure 2:**
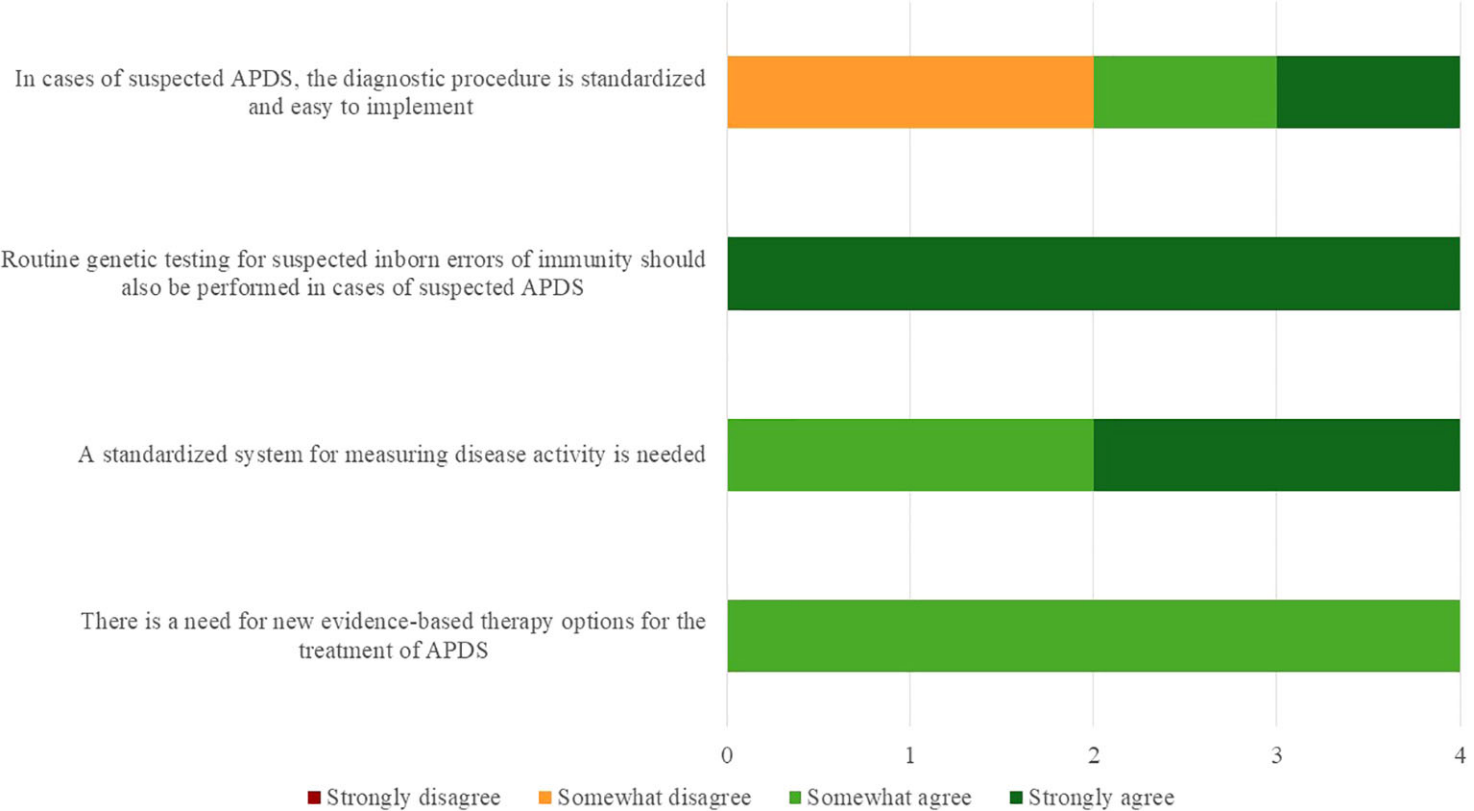
Statements validated by clinicians during the interviews.

At the end of the physician interviews, the four participants were asked whether they agreed or disagreed to a set of four hypotheses. The level of agreement was measured by a four-point Likert scale with the options to choose between “strongly disagree”, “somewhat disagree”, “somewhat agree” and “strongly agree”. The original, German, scale can be found as part of the interview guide as **Supplementary Material**. The following **Figure 2** summarizes the results of this assessment. While most respondents strongly agreed on the necessity of routine genetic testing for IEIs and supported the development of a standardized system for assessing APDS disease activity, opinions were more divided regarding the standardization and feasibility of current diagnostic procedures. Importantly, there was broad consensus on the urgent need for new evidence-based and targeted therapeutic options. Physicians noted that responses were made under the assumption that currently available therapies were not broadly accessible outside compassionate-use programs and confirmed that the perceived need would be addressed once targeted agents received formal approval.

## Discussion

This qualitative case study underscores the persistence of a prolonged diagnostic odyssey among individuals with APDS. Despite progress in molecular genetic testing, diagnostic delays remain the rule rather than the exception. The median delay of 11 years aligns with registry-based analyses and highlights the continuing gap between symptom onset and definitive diagnosis (17, 18).

Based on the recurring diagnostic patterns and barriers identified across patient and clinician interviews, a set of pragmatic red flags that may support earlier referral to immunology in clinical practice was derived:

1. Recurrent and atypical infections:
  Recurrent respiratory tract infections beginning in infancy or early childhood, particularly involving the upper airways and showing incomplete or short-lived responses to standard treatments, should raise suspicion of an underlying immunodeficiency.
2. Early-onset disease with multisystem involvement:
  Early symptom onset followed by a progressive disease course affecting multiple organ systems over time, including infectious, inflammatory, autoimmune, or lymphoproliferative manifestations, represents a key warning sign.
3. Lymphoproliferation and malignancy-related warning signs:
  Persistent lymphadenopathy, splenomegaly, or suspicion or occurrence of malignancy at a young age, especially in combination with recurrent infections or immune dysregulation, should prompt immunological evaluation.
4. Therapy-refractory inflammatory or autoimmune manifestations:
  Chronic inflammatory or autoimmune conditions, such as arthritis or eczema, that are refractory to standard immunosuppressive or anti-inflammatory therapies indicate possible underlying immune dysregulation.
5. Unexplained laboratory abnormalities:
  Incidental or unexplained immunological abnormalities, including hypogammaglobulinemia, or lymphocyte abnormalities that cannot be explained by isolated organ-specific disease, should trigger further immunological evaluation.

Published studies corroborate the findings of this study on the clinical presentation of patients with APDS, highlighting recurrent sinopulmonary infections, Crohn-like enteropathy, autoimmune cytopenia, growth delay, and bronchiectasis in older children and adolescents (4, 5, 12, 19, 20). Chronic respiratory infections occur in 85–95% of patients, lymphoproliferation in 86%, autoimmune phenomena in 13–56%, enteropathy in 30–45%, and bronchiectasis in up to 60% (6, 11, 21). Disease severity can vary even within families, and patients often undergo multiple evaluations before a correct diagnosis is considered. Therefore, the qualitative data is in line with current knowledge and validates our methodological approach.

According to the patients surveyed and the information provided by the physicians, the time to diagnosis and the interval between symptom onset and diagnosis is highly variable (22). Late referral to immunology is a major contributing factor, delaying diagnosis by an average of 10.5 years (23). Qualitative interviews confirmed that limited access to next-generation-sequencing historically posed barriers, and even today, many patients are initially seen by non-specialists who may not suspect an IEI. Early diagnosis often depends on chance encounters with knowledgeable clinicians (24). Structural improvements were proposed to address ongoing challenges, including enhanced interdisciplinary collaboration, streamlined referral pathways, and integration of immunologists into multidisciplinary teams. Models such as ambulatory specialized care (ASV) could be extended to immunology to improve continuity. Systematic education of non-immunologists and targeted awareness campaigns were emphasized as critical for early recognition and timely referral. Easy access to genetic testing with interpretable results was highlighted as a key intervention to accelerate diagnosis. Patients also noted the absence of structured support pathways beyond patient organizations. In total, even though necessary tools are available to make an early APDS diagnosis, structural implementation needs to be promoted.

## Conclusion

APDS is frequently diagnosed too late, prolonging morbidity and psychological burden. Early genetic testing, structured interdisciplinary care and timely access to targeted therapies can substantially improve outcomes. Health-system interventions that raise awareness and standardize pathways are critical to reducing the diagnostic odyssey.

## Limitations

This study is limited by the inherent subjectivity of data from patients, legal guardians, and physicians, which depends on participant input. The multi-perspective design combining patient and physician interviews mitigates bias through triangulation and provides contextualized insights. Analyses emphasized patterns with potential generalizability to the diagnostic odyssey of APDS, acknowledging the small sample sizes typical of rare disease research.

## Data Availability

The original contributions presented in the study are included in the article/**Supplementary Material**. Further inquiries can be directed to the corresponding author/s.
