# Supplementary material for: Diagnostic odyssey of patients with the rare immunodeficiency activated PI3 kinase delta syndrome (APDS): case study from expert and patient surveys
Source: Front Immunol. 2026 Mar 4;17:1763482. doi: 10.3389/fimmu.2026.1763482 (PMC12996251; doi:10.3389/fimmu.2026.1763482)
Supplement: Supplementary file 1 [file Table1.docx]

Interviewleitfaden

Diagnostische Odyssee von Patienten mit dem seltenen Immundeekt Activated PI3 Kinase Delta Syndrom (APDS)

Patienteninterviews

Erklärung zur Vertraulichkeit

Die in diesem Dokument enthaltenen Informationen sind vertraulich. Da es sich bei den Informationen um das geistige Eigentum der Pharming Group N.V. handelt, darf das Dokumente nicht ohne die Genehmigung der Pharming Group N.V. an Dritte weitergegeben werden. Die Informationen sind ausschließlich für den angegebenen Zweck der Durchführung einer Expertenbefragung von Ärzten und Patientenbefragung von APDS-Betroffenen zur Odyssee der Diagnosestellung eines APDS zu verwenden. Dies gilt auch für alle anderen zugehörigen Dokumente.

Hinweis

Im Folgenden wird entweder die weibliche oder die männliche Form verwendet, um den Lesefluss zu erleichtern. In allen Aussagen und Informationen sind dennoch alle Geschlechter enthalten, einschließlich weiblicher, männlicher und non-binärer Personen.

**Impressum**

Erstellt von

| 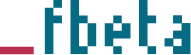 | fbeta GmbH  Akazienstraße 31  10823 Berlin  Deutschland |  |
| --- | --- | --- |

Sponsor

| 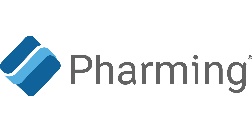 | Pharming Group N.V. Darwinweg 24  2333 CR Leiden  Niederlande |
| --- | --- |

# Qualitativer Fragebogen

Vielen Dank, dass Sie sich für diese Befragungsstudie zur Diagnosestellung des Aktivierenden PI3-Kinase-Delta-Syndroms (APDS) die erforderliche Zeit von etwa 20 Minuten Zeit nehmen.

Ziel dieser Befragung ist es, ein besseres Verständnis für die Erfahrungen von Patienten vom Auftreten erster Symptome, über die Diagnosestellung eines APDS, bis zur Einleitung einer Behandlung zu gewinnen. Hierbei sollen Arztgruppen identifiziert werden, die vor der definitiven Diagnosestellung schwerpunktmäßig in die Behandlung der Patienten involviert sind, um diese für APDS sensibilisieren zu können. Zugleich sollen die Faktoren herausgearbeitet werden, die maßgeblich für die Einleitung der Diagnostik waren, um betroffene Patienten mit APDS künftig schneller diagnostizieren und zielgerichteter behandeln zu können.

Die Befragung wird von der _fbeta GmbH im Auftrag der Pharming Group N.V. durchgeführt.

Ihre persönlichen Daten werden vertraulich behandelt, und die Aufzeichnungen werden anonymisiert. Die Interviewergebnisse werden nur für wissenschaftliche Zwecke verwendet.

Ihre Teilnahme an der Befragungsstudie ist freiwillig. Sie können jederzeit entscheiden, sich ohne die Angabe von Gründen zurückzuziehen, ohne dass dies negative Auswirkungen für Sie haben wird.

Sind Sie damit einverstanden, dass dieses Interview aufgenommen und die transkribierten Daten anonymisiert für wissenschaftliche Zwecke verwendet werden?

**Zeit von Symptomen bis zur Diagnosestellung eines APDS**

1. Wie alt waren Sie bzw. Ihr Kind zum Zeitpunkt der definitiven Diagnosestellung eines APDS?
2. Welche Krankheitsbeschwerden verursacht durch das APDS hatten Sie bzw. Ihr Kind vor und zum Zeitpunkt der definitiven Diagnosestellung?
3. Wurden Sie oder Ihr Kind vor der Diagnosestellung von Ärzten aufgrund Ihrer Symptome behandelt? Wenn ja, welchen Facharztgruppen gehörten die Ärzte an?
4. Haben Sie oder Ihr Kind nach Auftreten der ersten Anzeichen der Erkrankung eine spezifische Diagnose erhalten?
5. Wenn nicht, welche Faktoren führten Ihrer Ansicht nach dazu, dass Sie oder Ihr Kind nicht nach Auftreten der ersten Anzeichen der Erkrankung eine spezifische Diagnose bekommen haben?
6. Welcher Facharztgruppe gehört der Arzt an, welcher Ihnen bzw. Ihrem Kind die die definitive Diagnose eines APDS gestellt hat?
7. Welche Krankheitsbeschwerden haben Sie oder Ihr Kind aktuell auf Grund des APDS?
8. Hat sich Ihr Krankheitserleben bzw. das Krankheitserleben Ihres Kindes seit der Diagnosestellung eines APDS verändert?
   1. Welche Anpassungen des Lebensstils haben Sie vorgenommen?
   2. Wie haben sich die Symptome und die Auswirkungen der Erkrankung auf das tägliche Leben nach Einleiten der Therapie verändert?

**Therapie des APDS**

1. Welche Schritte wurden nach der Diagnosestellung eines APDS durch den behandelnden Arzt eingeleitet?
2. Inwiefern hat sich das Krankheitserleben seit Diagnosestellung verändert?
3. Welche Spezialisierungen haben Ärzte, welche an der Behandlung von Ihnen oder Ihrem Kind mit APDS beteiligt sind?
4. Welcher Facharztgruppe gehört der Arzt an, welcher bei Ihnen oder Ihrem Kind mit APDS die Koordination der Behandlung von weiteren Fachärzten und Heilberufen übernimmt?
5. Inwiefern sind Sie in die Entscheidung zur Auswahl einer geeigneten Therapieoption für Sie oder Ihr Kind mit APDS eingebunden?
6. Welche Termine nehmen Sie regelmäßig zur Kontrolle des APDS-Krankheitsverlaufs wahr?

**Herausforderungen bei der Diagnose und Behandlung des APDS**

1. Welche sind aus Ihrer Sicht die primären Herausforderungen bei der Diagnosestellung eines APDS?
2. Welche sind aus Ihrer Sicht die primären Herausforderungen bei der Therapie eines APDS?
3. Welche Voraussetzungen müssten geschaffen werden, um diesen Herausforderungen zu entgegnen?

Interviewleitfaden

Diagnostische Odyssee von Patienten mit dem seltenen Immundefekt Activated PI3 Kinase Delta Syndrom (APDS)

Experteninterviews

Erklärung zur Vertraulichkeit

Die in diesem Dokument enthaltenen Informationen sind vertraulich. Da es sich bei den Informationen um das geistige Eigentum der Pharming Group N.V. handelt, darf das Dokument nicht ohne die Genehmigung der Pharming Group N.V. an Dritte weitergegeben werden. Die Informationen sind ausschließlich für den angegebenen Zweck der Durchführung einer Expertenbefragung von Ärzten und Patientenbefragung von APDS-Betroffenen zur Odyssee der Diagnosestellung eines APDS zu verwenden. Dies gilt auch für alle anderen zugehörigen Dokumente.

Hinweis

Im Folgenden wird entweder die weibliche oder die männliche Form verwendet, um den Lesefluss zu erleichtern. In allen Aussagen und Informationen sind dennoch alle Geschlechter enthalten, einschließlich weiblicher, männlicher und non-binärer Personen.

**Impressum**

1. Erstellt von

| 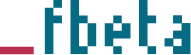 | fbeta GmbH  Akazienstraße 31  10823 Berlin  Deutschland |  |
| --- | --- | --- |

1. Sponsor

| 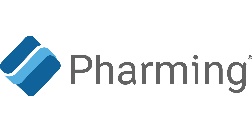 | Pharming Group N.V. Darwinweg 24  2333 CR Leiden  Niederlande |
| --- | --- |

- 1. **Qualitativer Fragebogen**

Vielen Dank, dass Sie sich für diese Befragungsstudie zur Diagnosestellung des Aktivierenden PI3-Kinase-Delta-Syndroms (APDS) die erforderliche Zeit von etwa 25 Minuten nehmen.

Ziel dieser Befragung ist es, ein besseres Verständnis für die diagnostischen Erfahrungen von Ärzten bei APDS zu gewinnen. Durch die gewonnenen Erkenntnisse soll der Verlauf vom Auftreten erster Symptome bis zur Diagnosestellung von APDS transparent gemacht werden. Hierbei sollen Arztgruppen identifiziert werden, die vor der definitiven Diagnosestellung schwerpunktmäßig in die Behandlung der Patienten involviert sind, um diese für APDS sensibilisieren zu können. Zugleich sollen die Faktoren herausgearbeitet werden, die maßgeblich für die Einleitung der Diagnostik waren, um betroffene Patienten mit APDS künftig schneller diagnostizieren und zielgerichteter behandeln zu können.

Die Befragung wird von der _fbeta GmbH im Auftrag der Pharming Group N.V. durchgeführt.

Ihre persönlichen Daten werden vertraulich behandelt, und die Aufzeichnungen werden anonymisiert. Die Interviewergebnisse werden nur für wissenschaftliche Zwecke verwendet.

Ihre Teilnahme an der Befragungsstudie ist freiwillig. Sie können jederzeit entscheiden, sich ohne die Angabe von Gründen zurückzuziehen, ohne dass dies negative Auswirkungen für Sie haben wird.

Sind Sie damit einverstanden, dass dieses Interview aufgenommen und die transkribierten Daten anonymisiert für wissenschaftliche Zwecke verwendet werden?

**Arztcharakteristika**

1. Welcher ärztlichen Fachgruppe gehören Sie an?
2. Haben Sie eine Zusatzweiterbildung zum Immunologen gemacht oder vergleichbare Kompetenzen erworben?
3. Inwiefern haben Sie durch Ihre ärztliche Tätigkeit Erfahrungen mit der Diagnosestellung und / oder Therapie von Patienten mit APDS?
4. Wie viele Patienten haben Sie in der Vergangenheit mit APDS behandelt und wie viele befinden sich aktuell bei Ihnen in Behandlung?

**Zeit von Symptomen bis zur Diagnosestellung des APDS**

1. Wie alt sind Patienten durchschnittlich zum Zeitpunkt der Diagnose eines APDS?
2. Wie weit fortgeschritten ist das APDS und die damit einhergehenden Symptome im Zeitraum vor und zum Zeitpunkt der definitiven Diagnosestellung?
3. Insofern zutreffend, welche Faktoren führen dazu, dass Patienten mit APDS nicht unmittelbar nach Auftreten der ersten Symptome die zutreffende Diagnose bekommen?
4. Wie lange dauert es durchschnittlich bis Patienten mit APDS nach Auftreten der ersten Symptome die zutreffende Diagnose bekommen?
5. Welche diagnostischen Verfahren werden für die Diagnosestellung des APDS verwendet?
6. Welche Spezialisierungen haben APDS-diagnostizierenden Ärzte?

**Therapie des APDS**

1. Welcher Facharztgruppen gehören die Ärzte an, welche die Krankheitsbeschwerden verursacht durch das APDS vor der Diagnosestellung behandelt haben?
2. Welche therapeutischen Schritte werden nach der Diagnose des APDS eingeleitet?
3. Wie lange dauert es durchschnittlich bis Patienten mit APDS nach Auftreten der ersten Symptome eine APDS-spezifische Therapie erhalten?
4. Wie verändert sich das Krankheitserleben der APDS-Patienten nach der Diagnosestellung?
   1. Welche Anpassungen des Lebensstils werden vorgenommen?
   2. Wie verändern sich die Symptome und die Auswirkungen der Erkrankung auf das tägliche Leben nach Einleiten der APDS-spezifischen Therapie?
5. Welche Spezialisierungen haben Ärzte, welche an der Behandlung von Patienten mit diagnostiziertem APDS beteiligt sind?
6. Welche Facharztgruppe übernimmt bei Kindern bzw. bei Erwachsenen mit APDS die Koordination einer multidisziplinären und multiprofessionellen Behandlung?
7. Inwiefern werden die APDS-Patienten in die Entscheidung zur Auswahl einer geeigneten Therapieoption eingebunden?
8. Inwiefern wird die Wahl einer angemessenen Therapieoption zur Behandlung des APDS durch Begleiterkrankungen der Patienten beeinflusst?
9. Wie wird der Krankheitsverlauf bezüglich Besserung und/oder Voranschreiten beurteilt?

**Herausforderungen bei der Diagnosestellung und Behandlung des APDS**

1. Welche sind aus Ihrer Sicht die primären Herausforderungen bei der Diagnosestellung des APDS?
2. Welche sind aus Ihrer Sicht die primären Herausforderungen bei der Therapie des APDS?
3. Welche Rahmenbedingungen müssten geschaffen werden, um diesen Herausforderungen zu entgegnen?
   1. **Quantitative Fragen für Onlinebefragung**

Vielen Dank, dass Sie sich für diese Onlinebefragung zur Diagnosestellung des Aktivierenden PI3-Kinase-Delta-Syndroms (APDS) die erforderliche Zeit von ca. 5 Minuten Zeit nehmen.

Ziel dieser Onlinebefragung ist es ergänzende Informationen zusätzlich zu den Interviews zu gewinnen. Durch die gewonnenen Erkenntnisse soll der Verlauf vom Auftreten erster Symptome bis zur Diagnosestellung von APDS transparent gemacht werden. Hierbei sollen Arztgruppen identifiziert werden, die vor der definitiven Diagnosestellung schwerpunktmäßig in die Behandlung der Patienten involviert sind, um diese für APDS sensibilisieren zu können. Zugleich sollen die Faktoren herausgearbeitet werden, die maßgeblich für die Einleitung der Diagnostik waren, um betroffene Patienten mit APDS künftig schneller diagnostizieren und zielgerichteter therapieren zu können.

Die Befragung wird von der _fbeta GmbH im Auftrag der Pharming Group N.V. durchgeführt.

Ihre persönlichen Daten werden vertraulich behandelt und nur für wissenschaftliche Zwecke verwendet.

Ihre Teilnahme an der Onlinebefragung ist freiwillig. Sie können jederzeit entscheiden, sich ohne die Angabe von Gründen zurückzuziehen, ohne dass dies negative Auswirkungen für Sie haben wird.

**Fragen zur Diagnosestellung des APDS in Deutschland**

1. Wie hoch schätzen Sie die Gesamtanzahl an diagnostizierten Patienten mit APDS in Deutschland?
2. Wie hoch schätzen Sie den Anteil der Patienten mit APDS an der Gesamtanzahl in Deutschland, welche keine explizite APDS-Diagnose erhalten haben?
3. Wie lange dauert es durchschnittlich vom Auftreten erster Symptome bis ein Patient mit APDS eine definitive Diagnose bekommt?

**Hypothesen zur Diagnose und Therapie des APDS in Deutschland**

Bei Verdacht auf APDS ist das diagnostische Verfahren standardisiert und gut umzusetzen.

____________________________________________________________

Stimme nicht zu Stimme eher nicht zu Stimme eher zu Stimme zu

Die routinemäßige genetische Testung bei Verdacht auf primäre Immundefekte sollte auch bei Verdacht auf APDS durchgeführt werden.

____________________________________________________________

Stimme nicht zu Stimme eher nicht zu Stimme eher zu Stimme zu

Es bedarf einer einheitlichen Systematik zur Messung der Krankheitsaktivität.

____________________________________________________________

Stimme nicht zu Stimme eher nicht zu Stimme eher zu Stimme zu

Es bedarf neuer evidenz-basierter Therapieoptionen für die Behandlung eines APDS.

____________________________________________________________

Stimme nicht zu Stimme eher nicht zu Stimme eher zu Stimme zu
